# Glabridin-induced vasorelaxation: Evidence for a role of BK_Ca_ channels and cyclic GMP

**DOI:** 10.1016/j.lfs.2016.09.018

**Published:** 2016-11-15

**Authors:** Debrabata Chanda, Jesus Prieto-Lloret, Arjun Singh, Hina Iqbal, Pankaj Yadav, Vladimir Snetkov, Philip I Aaronson

**Affiliations:** a Division of Asthma, Allergy & Lung Biology, School of Medicine, King's College London, London WC2R 2LS, United Kingdom; b In-vivo Testing Facility, Molecular Bioprospection Department, Biotechnology Division CSIR-Central Institute of Medicinal and Aromatic Plants, Lucknow, 226015, Uttar Pradesh, India

**Keywords:** BK_Ca_ channels, Cyclic GMP, Glabridin, Mesenteric artery, Rat

## Abstract

**Background and purpose:**

Glabridin is a major flavonoid in *Glycyrrhiza glabra* (licorice) root, a traditional Asian medicine. Glabridin is reported to have anti-atherogenic, anti-inflammatory and anti-nephritic properties; however its effects on vascular tone remain unexplored.

**Experimental approach:**

We examined the effect of glabridin on rat main mesenteric artery using isometric myography and also ELISA to measure cGMP levels.

**Key results:**

Glabridin (30 μM) relaxed arteries pre-constricted with the thromboxane A_2_ analog U46619 (0.2 μM) by ~ 60% in an endothelium-independent manner. Relaxation to 30 μM glabridin was abolished by the guanylate cyclase inhibitor 1H-[1,2,4]oxadiazolo[4,3-a]quinoxalin-1-one (1 μM) and by the BK_Ca_ channel blocker tetraethyammonium (1 mM) but was unaffected by the estrogen receptor antagonist ICI182780. The concentration-response curve to glabridin (0.1 to 30 μM) was downshifted by the K_ATP_ channel blocker glibenclamide (10 μM), the K_V_ channel blocker 4-aminopyridine (300 μM), and the K_IR_ blocker BaCl_2_ (30 μM). In U46619-contracted arteries partially relaxed by 0.1 μM sodium nitroprusside, application of 10 and 30 nM glabridin caused additional vasorelaxation. Glabridin (30 μM) approximately doubled tissue [cyclic GMP]. Application of the phosphodiesterase inhibitor isobutylmethylxanthine caused a much larger rise in [cyclic GMP], and glabridin failed to cause vasorelaxation or a further rise in [cGMP] when co-applied with IBMX.

**Conclusions and implications:**

Vasorelaxation to glabridin is dependent on the opening of K^+^ channels, particularly BK_Ca_, probably caused by a rise in cellular [cyclic GMP] owing to phosphodiesterase inhibition. In the presence of sodium nitroprusside an effect of glabridin is observed at nM concentrations, similar those measured in plasma following human ingestion of licorice flavonoid oil.

## Graphical abstract

**Figure:**
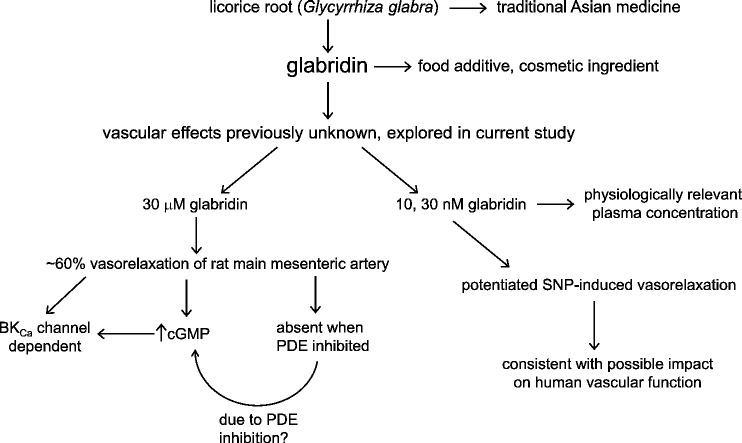

## 1 Introduction

The isoflavone glabridin is an important constituent of licorice root (*Glycyrrhiza glabra*), from which it was first isolated in 1976 [1]. Preparations containing *Glycyrrhiza glabra* have been used by Indian and Chinese medicine for centuries as a treatment for many diseases, including ulcers, bronchitis and bacterial infections, and glabridin has anti-oxidant, anti-inflammatory and skin-whitening properties, providing a rationale for its inclusion in many cosmetic preparations. Moreover, Glavonoid®_,_ a standardised polyphenol extract of licorice root containing 3% glabridin, is being marketed as a food supplement and anti-obesity agent, and is added to yoghurt and fruit juices in many Western countries.

As described in a recent review by Simmler and colleagues [2], a burgeoning literature comprising studies in vitro and in animals in vivo has shown that glabridin has anti-inflammatory, anti-atherogenic, estrogenic, neuroprotective, and anti-osteoporetic actions. It is however unclear to what extent these effects contribute to the putative beneficial actions of licorice-derived preparations because most studies have employed micromolar concentrations of glabridin, whereas its likely plasma concentration range in humans following ingestion of licorice-derived products is likely to be in the low nanomolar range [2]. An exception to this, however, is the glabridin-induced activation of estrogen receptors in vascular smooth muscle, which has been observed to occur at concentrations as low as 10 nM [3].

In view of the widespread and long-standing use of preparations containing *Glycyrrhiza glabra* and the increasing use of glabridin-enriched extracts as food supplements and ingredients, it is noteworthy that very little is known about its acute effects on the vasculature. Moreover, Tominaga et al. (2009) have demonstrated that ingestion of glabridin for 8 weeks significantly reduced BMI and visceral fat in human volunteers [4], implying that glabridin might be useful as a treatment for obesity. Considering that obesity is very common in those with hypertension and coronary heart disease, it seems likely that the ingestion of glabridin will increase in such patients, in which case it is important that potential effects of this agent on the cardiovascular system be understood. We therefore examined the effect of glabridin in rat main mesenteric artery using a small vessel isometric myograph, and report here for the first time that glabridin acts as a cGMP-dependent vasodilator.

## 2 Methods

### 2.1 Animal welfare and ethical statement

This study conforms with the *Guide for the Care and Use of Laboratory Animals* published by the US National Institutes of Health (NIH Publication No. 85–23, revised 1996) and is in accordance with UK Animals (Scientific Procedures) Act, 1986, Amendment Regulations (SI 2012/3039). Male Wistar rats (200–300 g; 6–8 weeks old) were killed by lethal injection (*i.p.*) of sodium thiopental.

### 2.2 Mounting of mesenteric arteries and measurement of tension development

Intestines were excised and the main mesenteric artery and its primary branches were removed, placed in physiological salt solution (PSS), containing in mM: NaCl 118; NaHCO_3_ 24; KCl 4; CaCl_2_ 1.8; MgSO_4_ 1; NaH_2_PO_4_ 0.43; and glucose 5.56, and the main mesenteric artery was freed of surrounding adipose tissue, cut into rings and mounted on a conventional small vessel wire myograph (Danish Myo Technology, Aarhus, Denmark). Tension was recorded at a frequency of 1 Hz. Mesenteric artery rings were stretched to give a basal tension of 2–3 mN, and were equilibrated with three brief exposures to PSS containing 80 mmol/l KCl (80KPSS; isotonic replacement of NaCl by KCl). 0.2 μM U46619 was then applied to give a near maximal contraction, and endothelial integrity was confirmed by recording vasorelaxation to 10 μM carbachol. Following washout, arteries were again constricted, with either 0.2 μM U46619 or 10 μM norepinephrine (NE). When the tension had reached a maximum, the vasorelaxing effect of glabridin was recorded as described in the Results. Where appropriate, the endothelium was removed by rubbing the vascular lumen with a human hair, and its functional absence was confirmed by the absence of carbachol-induced relaxation. All drugs used in these studies were sourced from Sigma-Aldrich, UK).

In order to study the concentration-dependency of glabridin-induced vasorelaxation, the contraction to U46619 (or NE, when the effect of glibenclamide was being studied) was allowed to reach its maximum, and then ascending concentrations of glabridin, (0.1–30 μM), were applied cumulatively at 15 min intervals, with the effect of glabridin being recorded at the end of each interval. Because the contractions to U46619 and NE demonstrated some intrinsic rundown, each arterial ring in which glabridin was studied was paired with an arterial segment from the same animal in which the amplitude of the contraction to U46619 or NE was recorded in the absence of glabridin at the same times at which contraction was measured in the presence of glabridin (i.e. a time control). The percent relaxation caused by glabridin was then calculated as 100 × ((1 − A) − (1 − B)) / (1 − A), where A was the percent relaxation recorded at a given time in the time control segment and B was the percent relaxation observed at the same time in the paired segment treated with glabridin.

### 2.3 Cyclic GMP measurements

Intracellular cyclic GMP (cGMP) in rat mesenteric arteries was measured as previously described [5] with minor modifications. Endothelium-intact arterial segments [approx. 5 mg each (wet weight)] were equilibrated in PSS for 30 min at 37 °C under continuous carbogen bubbling and were then exposed to U-46199 (200 nM; 10 min), glabridin (30 μM; 20 min), U46199 (200 nM) followed by glabridin (30 μM; 20 min), IBMX (200 μM; 30 min) and IBMX (200 μM; 10 min) followed by glabridin (30 μM; 20 min). DMSO (3 μl; 10 min) was added as a vehicle control to a further group of tissues. Tissues were snap frozen in liquid nitrogen immediately after treatment. The frozen tissues were pulverized in a pre-chilled (with liquid nitrogen) pulverizer. The tissue pellet was suspended in chilled 5% trichloro acetic acid (200 μl) and vortexed vigorously and then centrifuged at 5000*g* for 10 min at 8 °C. Supernatant thus obtained was extracted five times with water-saturated diethyl ether and was then used for cGMP assay, using an ELISA kit (Cayman Chemical Company, Ann Arbor, MI, USA), following the manufacturer's instructions. Measurements were made in 3 aliquots from each tissue, and resulting values were averaged to produce each data point used in subsequent calculations. Tissue pellets obtained after centrifugation were dissolved in 0.5 N NaOH for protein estimation by the BCA method and the concentration of cGMP was expressed as picomoles/mg protein.

### 2.4 Data analysis and statistical procedures

Results were generally analyzed using Student's unpaired or paired t-test, as appropriate, with p < 0.05 taken to indicate statistical significance. Results are shown as mean ± SEM. The effects of antagonists on the galbridin concentration-response relationship shown in Fig. 4 were analyzed using repeated measures ANOVA with a Bonferroni post hoc test. GraphPad Prism version 7 was used for statistical analysis and preparation of figures.

## 3 Results

**Fig. 1:**
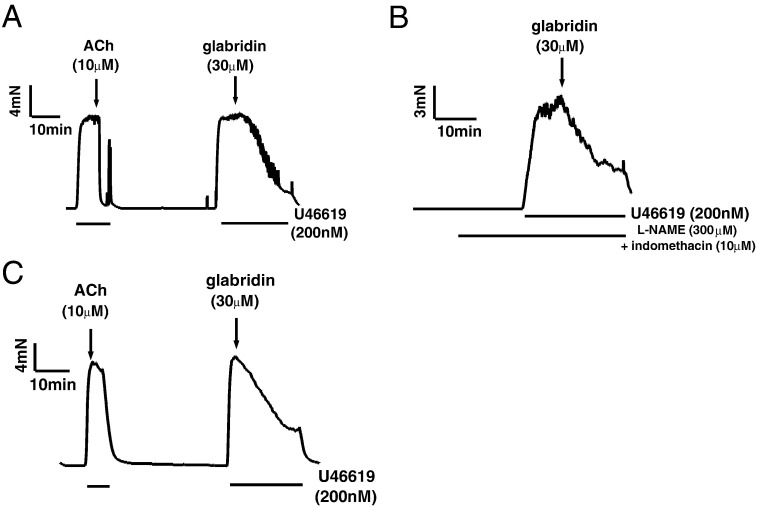
Lack of involvement of the endothelium in glabridin-induced vasorelaxation. Representative traces illustrating the relaxation by 30 μM glabridin of mesenteric arteries preconstricted with 0.2 μM U46619. A) endothelium-intact artery, B) endothelium-intact artery pretreated with 300 μM L-NAME and 10 μM indomethacin, C) endothelium-denuded artery.

Initial experiments revealed that 30 μM glabridin caused a rapid and robust relaxation of mesenteric arteries constricted with 0.2 mM U46619. Fig. 1A illustrates an example of the vasorelaxing effect of 30 μM glabridin in endothelium-intact mesenteric arteries (note the endothelium-dependent relaxation of the U46619 contracture to 10 μM acetylcholine; ACh). Similar responses to glabridin were observed in endothelium-intact mesenteric arteries which had been pre-treated with 300 μM L-NAME and 10 μM indomethacin to prevent the synthesis of nitric oxide (NO) and prostacyclin (PGI_2_) respectively (Fig. 1B) and also in the absence of endothelium (Fig. 1C). These data indicated that the endothelium played no role in mediating glabridin-induced relaxation.

**Fig. 2:**
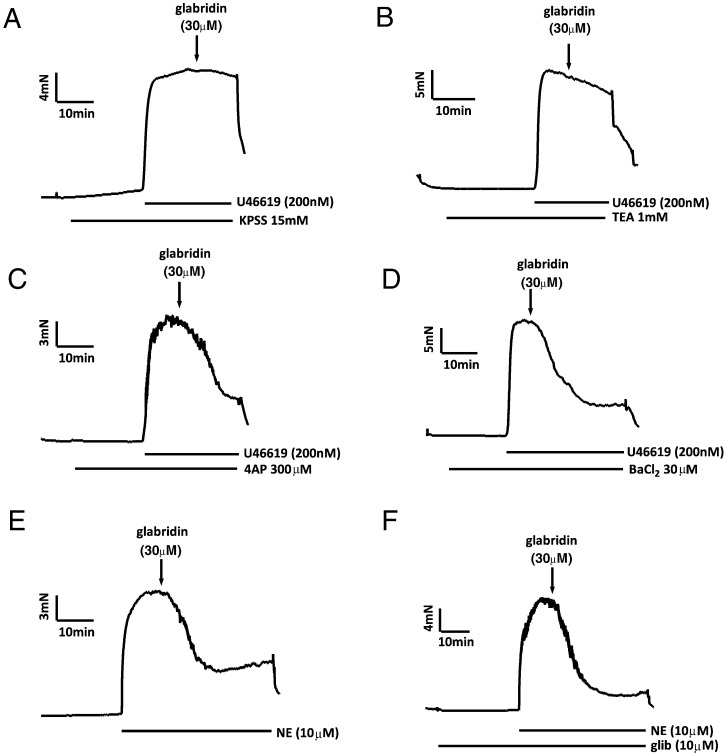
Effects of 15 mM K^+^ and K^+^ channel antagonists on glabridin-induced vasorelaxation. Representative traces illustrating the effect of 30 μM glabridin on the U46619-induced contraction in the presence of (A) PSS containing 15 mM K^+^, (B) 1 mM TEA, (C) 300 μM 4-AP, and (D) 30 μM Ba^2 +^. E) Representative traces showing the effect of 30 μM glabridin on the norepinephrine-induced contraction in the absence (left) and presence (right) of 10 μM glibenclamide.

Fig. 2 presents evidence that relaxation induced by 30 μM glabridin was strongly dependent on the opening of large-conductance Ca^2+^ activated K^+^ (BK_Ca_) channels. As shown in Fig. 2A, glabridin caused no relaxation of U46619-constricted arteries in PSS in which the [K^+^] had been raised to 15 mM in order suppress any hyperpolarization (and resulting relaxation) associated with K^+^ channel opening. Glabridin-induced relaxation was also strongly inhibited in the presence of the BK_Ca_ channel blocker tetraethylammonium (TEA; 1 mM) (Fig. 2B). Conversely, neither the K_V_ channel antagonist 4-aminopyridine (4-AP; 300 μM) nor the K_IR_ channel blocker BaCl_2_ (30 μM) had any significant effect on the response to glabridin (Fig. 2C and D, respectively). The K_ATP_ channel blocker glibenclamide was used in order to determine whether these channels were involved in relaxation induced by 30 μM glabridin. Because glibenclamide blocks contractions induced by U46619 [6], NE (10 μM) was used to elicit pre-constriction in these experiments. As shown in Fig. 2E and F, glabridin-induced relaxation of the norepinephrine contraction, which was similar to that observed when U46619 was used to cause preconstruction, was unaffected by glibenclamide.

**Fig. 3:**
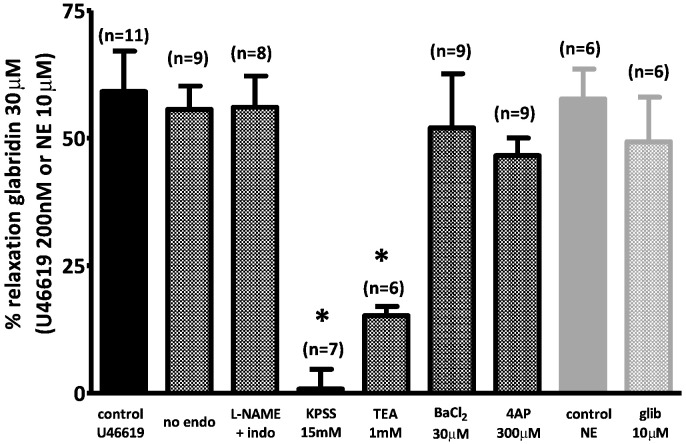
Mean effect of 30 μM glabridin under control conditions and following interventions designed to block endothelium–dependent or K^+^ channel-mediated vasorelaxation. Mean ± SEM percent relaxation induced by 30 μM glabridin under the various conditions illustrated by the traces in Fig. 1, Fig. 2. Asterisks indicate that the effect of glabridin under a particular condition was significantly (p < 0.05) different than that recorded under control conditions (i.e. endothelium-intact mesenteric arteries preconstricted with 0.2 μM U46619). Each bar represents the results of 6–11 experiments.

Fig. 3 presents the mean effects of endothelial denudation, the combination of L-NAME and indomethacin, 15 mM K^+^ PSS, and the K^+^ channel blockers on the vasorelaxation measured 10 min after the application of 30 μM glabridin.

Although the results shown in Fig. 2, Fig. 3 indicate that BK_Ca_ channels play an overriding role in the response to 30 μM glabridin, they do not rule out the possibility that other K^+^ channels might be involved in the vasorelaxation caused by lower glabridin concentrations. We therefore examined the concentration-dependency of glabridin-mediated vasorelaxation in the presence and absence of the K^+^ channel blockers TEA, 4-AP, Ba^2 +^ and glibenclamide. We similarly examined the effect of 300 μM L-NAME and 10 μM indomethacin on the glabridin concentration-response relationship.

**Fig. 4:**
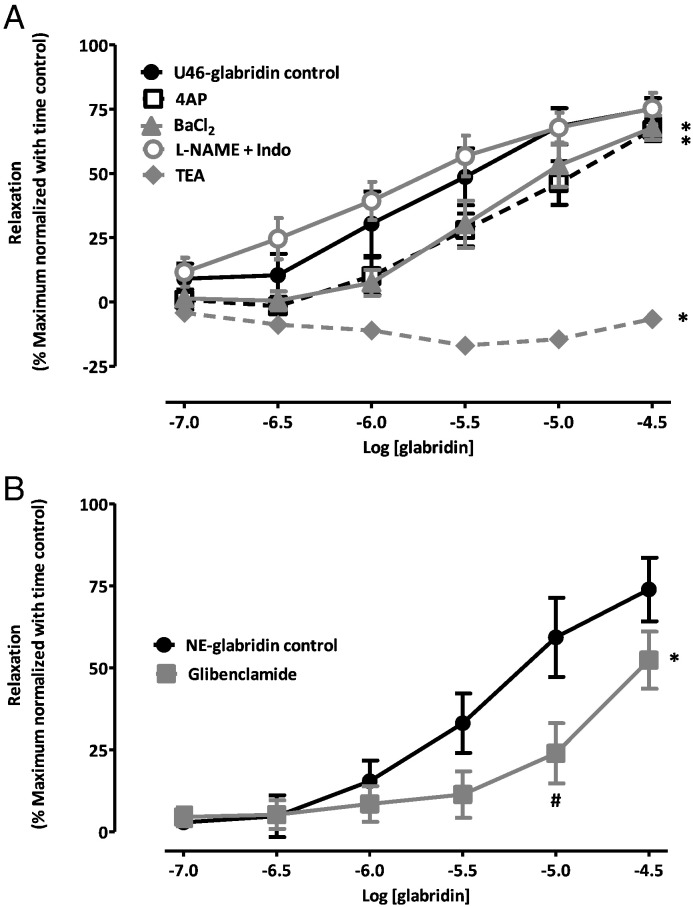
Effects of blockers on the concentration-response curve for glabridin vasorelaxation. (A) Mean ± SEM percent relaxation of the contraction to 0.2 mM U46619 induced by 0.1–30 μM glabridin under control conditions and in the presence of 300 μM L-NAME and 10 μM indomethacin, 1 mM TEA, 300 μM 4-AP, and 30 μM Ba^2 +^. (B) Mean ± SEM percent relaxation of the contraction to 10 μM NE induced by 0.1–30 μM glabridin under control conditions and in the presence of 10 μM glibenclamide. For both panels, * to the right of the point at 30 μM glabridin indicates a significant inhibition of the response to glabridin over the range of concentrations examined, whereas ^#^ adjacent to a point on the curve indicates a significant effect of the blocker at that concentration of glabridin. Vasorelaxation was corrected for intrinsic time-dependent rundown of the contractile response as described in the Methods. Each curve represents the results of 5–8 experiments.

As shown in Fig. 4, whereas the combination of L-NAME and indomethacin had no significant effect on the glabridin concentration-response curve, 4-AP, Ba^2 +^ (Fig. 4A) and glibenclamide (Fig. 4B) appeared to generally suppress vasorelaxation at lower glabridin concentrations. This attenuation of the response to glabridin was not significant for individual concentrations of any of these three blockers with the exception of the effect of glibenclamide on the response to 10 μM glabridin. However the effect on the entire concentration-response curve for each of these blockers, as assessed using repeated measures ANOVA, was highly significant. TEA, which had almost abolished the response to 30 μM glabridin, had a similar effect over the entire range of concentrations tested (Fig. 4A).

**Fig. 5:**
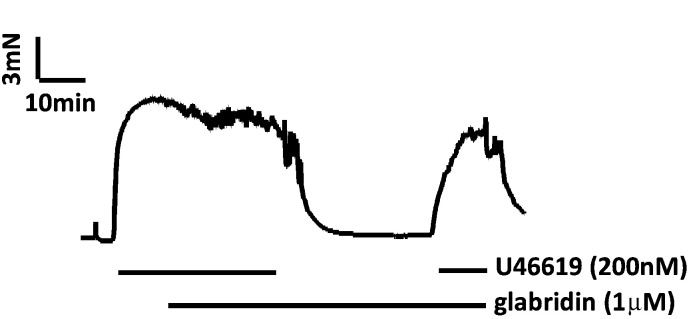
Effect of pretreatment with 1 μM glabridin on the amplitude of the U46619 contraction. Representative trace illustrating the effect of 60 min pretreatment with 1 μM glabridin on the contraction evoked by 0.2 μM U46619.

Although ≤ 1 μM glabridin often appeared to cause a concentration-dependent vasorelaxation, this effect was not statistically significant. This may have been because its effect at these low concentrations was slow and therefore difficult to unambiguously distinguish from the gradual intrinsic decay of the U46619 contraction. In order to determine whether the possible effect of 1 μM glabridin on tension was real, we therefore used a pre-treatment protocol, as illustrated in Fig. 5. In these experiments, glabridin was applied at the peak of the U46619 contraction. 30 min later, U46619 and glabridin were washed out of the bath, and 1 μM glabridin was immediately re-applied. Following a further 30 min incubation in glabridin, U46619 was again applied, and the peak contraction was recorded. As shown in Fig. 5, the second U46619 contraction was smaller than the first. In five similar experiments, the amplitude of the U46619 contraction fell by 27.0 ± 6.8% in the presence of glabridin (from 12.7 ± 1.4 to 9.4 ± 1.4 mN, p < 0.05 by paired *t*-test).

**Fig. 6:**
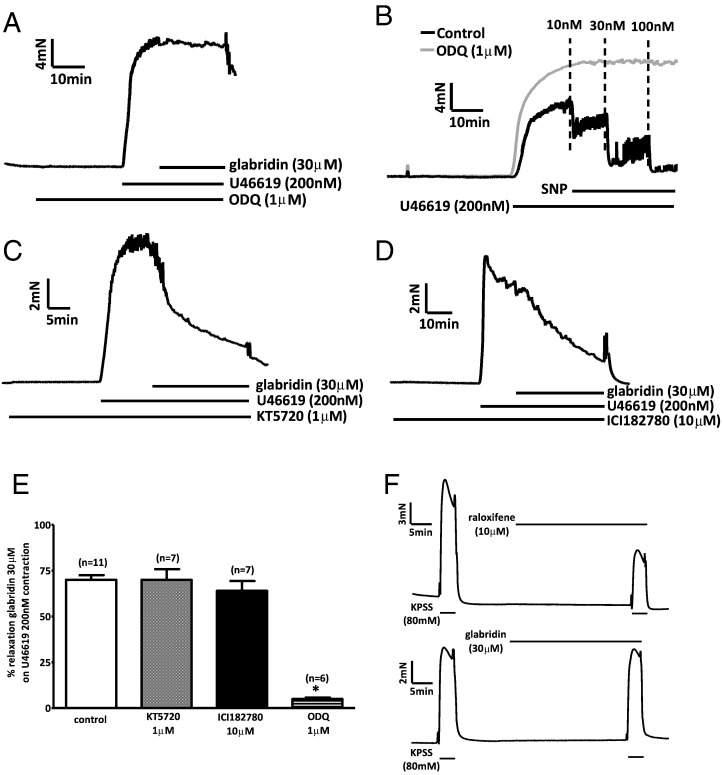
Effects of ODQ, KT5720 and ICI 182780 on vasorelaxation of the contraction to 200 nM U46619 by 30 μM glabridin. A) Representative trace showing the effect of glabridin in the presence of 1 μM ODQ. B) Representative traces showing the effect of SNP in the absence (black line) and presence (grey line) of 1 μM ODQ. Representative traces showing the effect of glabridin in the presence of KT5720 (C; 1 μM) and ICI 182780 (D; 10 μM). E) Average relaxation of the U46619 contraction by glabridin in the absence of another drug, and in the presence of KT5720, ODQ, and ICI 182780). Numbers of experiments used to calculate the results are shown over the bars, and asterisks represent a significant difference vs the control relaxation. (F) Representative traces depicting the effect on the contraction evoked by 80KPSS of preincubation of arteries with 10 μM raloxifene (top) or 30 μM glabridin (bottom).

Cyclic nucleotides are known to open BK_Ca_ channels present in vascular smooth muscle. As shown in Fig. 6A and E, the guanylate cyclase inhibitor 1H-[1,2,4]oxadiazolo[4,3-a]quinoxalin-1-one (ODQ; 1 μM) abolished the effect of 30 μM glabridin. The efficacy of this concentration of ODQ against guanylate cyclase was evidenced by its ability to ablate vasorelaxation elicited by the NO donor SNP (Fig. 6B, E). On the other hand, the protein kinase A antagonist KT-5720 (1 μM, Fig. 5C) and the estrogen receptor blocker ICI 182780 (10 μM; Fig. 6D, E) had no effect on the relaxation to 30 μM glabridin. Further evidence that glabridin was not acting via estrogen receptors is presented in Fig. 6F. Whereas treatment of tissues with the selective estrogen receptor modulator raloxifene (10 μM) attenuated the contraction induced by depolarization with 80 mM KPSS (49.5), glabridin (30 μM) did not affect the response to high K^+^ (2.4 ± 3.3% decrease, n = 4, ns vs control, p < 0.05 vs effect of raloxifene).

**Fig. 7:**
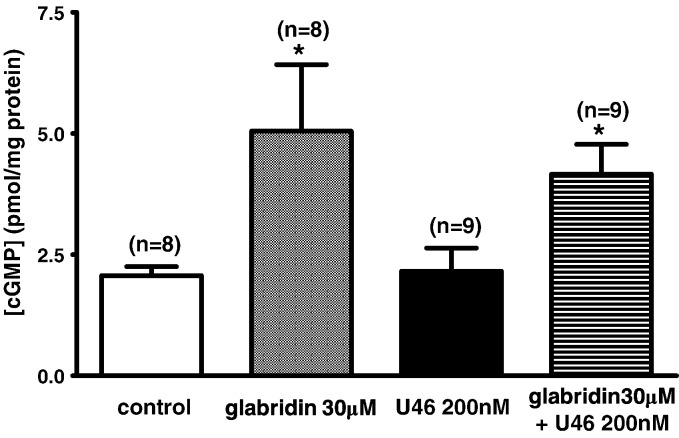
Effects of 30 μM glabridin on cGMP levels in rat mesenteric artery segments in the presence and absence of U46619. * indicates a significant effect of glabridin on [cGMP] compared to the corresponding control. Numbers of experiments (i.e. measurements in individual artery segments) used to calculate the results are shown over the bars, and asterisks represent a significant difference vs the control cGMP level.

The results described above were consistent with the possibility that glabridin was causing vasorelaxation via a cGMP-dependent activation of K^+^ channels. In this case, it would be predicted that glabridin should increase the cellular concentration of cGMP. As illustrated in Fig. 7A, a 15 min treatment with 30 μM glabridin caused a significant increase in the cyclic GMP content of segments of mesenteric artery, which was similar in the presence and absence of 200 nM U46619, which itself had no effect on the cGMP content.

The two most straightforward possible explanations for the ability of glabridin to raise the cGMP content of the arteries were that it was either stimulating guanylate cyclase or inhibiting phosphodiesterase (PDE). In this case, we reasoned that if glabridin was acting as a PDE inhibitor it would exert no effects on vascular tone or tissue cGMP if PDE was already maximally inhibited. Conversely, if glabridin was acting by stimulating guanylate cyclise, its effects on vascular tone and [cGMP] should persist (and might even be potentiated) if it was applied when PDE was already blocked. We therefore examined the effects of glabridin in the presence of a high concentration (200 μM) of the non-selective cGMP PDE inhibitor isobutylmethylxanthine (IBMX).

**Fig. 8:**
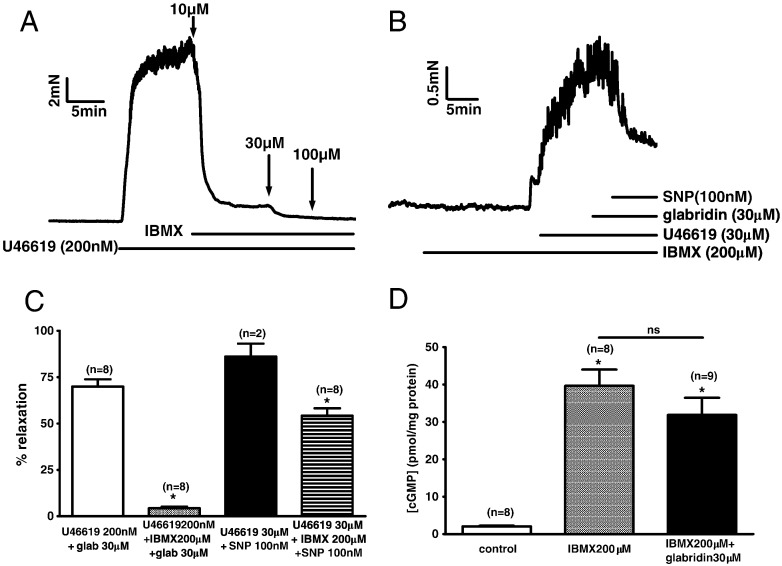
Effect of IBMX on glabridin-induced responses. A) Representative trace showing the effect of IBMX on the contraction induced by 200 nM U46619. B) Trace illustrating the effect of sequential addition of 30 μM glabridin and 100 nM SNP on the contraction evoked by 30 μM U46619 in the presence of 200 μM IBMX. C) Average vasorelaxation elicited by 30 μM glabridin when applied in the presence of 200 nM U46619 or 200 μM IBMX and 200 nM U46619, and vasorelaxation to 300 nM SNP when applied in the presence of 200 nM U46619 or 200 μM IBMX and 200 nM U46619. * indicates a significant block of the response to glabridin or SNP in the presence of IBMX. D) Effects of 200 mM IBMX on cGMP levels in rat mesenteric artery segments in the absence and presence of 30 μM glabridin. * indicates a significant increase in cGMP content compared to control. In panels C and D, numbers of experiments (i.e. measurements from individual arterial segments) used to calculate the results are shown over the bars.

Fig. 8A shows that treatment with 100 μM IBMX was enough to virtually abolish the U46619 contraction to 200 nM U46619. However, even in the presence of a somewhat higher concentration of IBMX (200 μM), it was possible to evoke a contraction which was of sufficient amplitude to be measured accurately if a very high concentration of U46619 (30 μM) was used (Fig. 8B). This residual contraction was unaffected by the application of 30 μM glabridin (Fig. 8B). On the other hand, subsequent application of the nitric oxide donor SNP (300 nM), which would be expected to stimulate guanylate cyclase, caused a marked vasodilation. These results are summarised in Fig. 8C. Fig. 8D illustrates the effect of 200 μM IBMX and the combination of 200 μM IBMX and 30 μM glabridin on the cGMP content of segments of mesenteric artery. IBMX caused the expected large rise in [cGMP], but glabridin exerted no further effect.

**Fig. 9:**
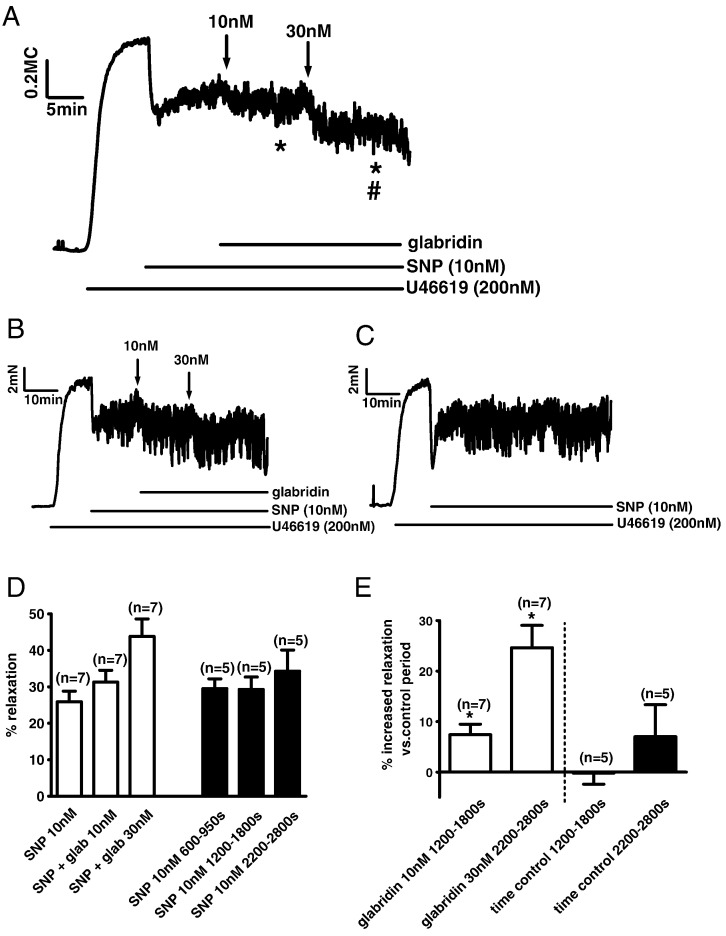
Glabridin (10 and 30 nM) caused further relaxation of the contraction to 200 nM U46619 in the presence of 0.1 μM SNP. (A) Arteries were contracted with 200 nM U46619 and then 0.1 μM SNP was applied to cause a partial relaxation. Subsequent application of 10 and then 30 nM glabridin caused further relaxation. The line represents the average of seven individual experiments. * indicates a significant fall in the average tension level during the final 120 s of the periods during which 10 and 30 nM glabridin were present, compared to the final 120 s of the period just prior to glabridin application, and ^#^ indicates a significant difference between the average tension recorded during these intervals in the presence of 10 and 30 nM glabridin. (B) Representative trace from one of the seven experiments from which the mean data in panel A is derived. (C) Representative trace of a time control experiment, in which SNP but not glabridin was applied during the response to U46619. (D) The white bars represent the mean ± SEM % relaxation observed in the presence of SNP alone, and in SNP following the application of 10 nM and then 30 nM glabridin, calculated using the average tension recorded 350–600, 1200–1800 and 2200–2800 after the application of SNP, respectively. The black bars represent the % relaxation recorded in five parallel time control experiments over the same time intervals after the application of SNP alone. (F) The white bars show the mean ± SEM change in % relaxation induced by 10 nM and 30 nM glabridin compared to SNP alone. The black bars show the mean ± SEM change in % relaxation which occurred during the corresponding time intervals when SNP but not glabridin was present. * indicates a significant difference between the % relaxation in the presence and absence of 10 and 30 nM glabridin.

In light of the possibility that glabridin might be acting as a PDE inhibitor, we reasoned that the vasorelaxing effects of low concentrations of glabridin might be potentiated if cGMP levels were first elevated by the NO donor sodium nitroprusside (SNP). We therefore applied 100 nM SNP at the peak of the U46619 contraction to evoke an immediate relaxation of 30–40%, and then applied 10 and 30 nM glabridin. Tension levels in the presence of glabridin were compared to tension during the same period in control tissues in which only SNP was added. Results from 7 tissues treated with glabridin were normalized and averaged, and are illustrated in Fig. 9A. It is evident that whereas the level of tension tended to recover slightly in the presence of SNP only, tension fell in a stepwise manner after application of 10 and 30 nM glabridin. Fig. 9B and C depict individual experiments in which glabridin was either applied or not applied after SNP. Fig. 9D represents the mean relaxation observed when the effects of SNP or SNP + glabridin had stabilized (left), or during the same time intervals in arteries to which only SNP had been added (right). Fig. 9E shows the mean increase in relaxation between these time periods in the presence and absence of glabridin. Glabridin relaxed the arteries significantly at both 10 and 30 nM, whereas no significant time-dependent relaxation was observed with SNP alone; moreover this difference was significant. These data indicate that glabridin exerts a small but measurable effect on mechanisms controlling vascular tone at concentrations which are relevant to the use of this drug as a possible therapeutic agent [7].

## 4 Discussion and conclusions

Glabridin has been shown to have several effects which could be beneficial for the cardiovascular system, although the extent to which these contribute to the putative therapeutic actions of licorice root is unclear. Notably, in vitro and in vivo studies show that glabridin inhibits low density lipoprotein oxidation and atherogenesis [8], possibly by inhibiting NADPH oxidase [9] or by increasing the expression of antioxidant enzymes in macrophages [10]. Somjen et al., (2004) also showed that 30 nM glabridin caused DNA synthesis and a stimulation of creatine kinase activity in an endothelial cell line (ECV-304) and cultured uterine artery smooth muscle cells [3]. Both effects were blocked by the estrogen receptor antagonist raloxifene. Moreover, glabridin has been shown to reduce adiposity in obese rodents, an action which may result from an inhibition of mitochondrial ATP synthesis with a consequent stimulation of AMP kinase [11].

Our observations indicate that glabridin evokes vasorelaxation by opening K^+^ channels, an effect which would be predicted to cause vascular smooth muscle cell hyperpolarization, resulting in the closure of voltage-gated Ca^2 +^ channels and, as a consequence, a fall in [Ca^2 +^]. This would explain the abolition of the response to glabridin in PSS containing 15 mM K^+^, at which concentration smooth muscle hyperpolarization beyond − 50 mV should be prevented [12]. The virtual abolition of the glabridin-induced relaxation by TEA, which is relatively selective for BK_Ca_ channels at the concentration we employed (1 mM) [13], indicates a predominant role for this type of K^+^ channel. However, relaxation to lower concentrations of glabridin was also attenuated by blockers of voltage-gated (K_V_), inwardly rectifying (K_IR_) and ATP-sensitive (K_ATP_) K^+^ channels, suggesting that the opening of these channels was reinforcing BK_Ca_-induced hyperpolarization.

Relaxation was blocked by the guanylate cyclase inhibitor ODQ, indicating that glabridin-induced relaxation was likely occurring via cGMP. This possibility was supported by the finding that glabridin caused an approximately two-fold increase in the cGMP content of arterial segments. There is abundant evidence that raising VSMC cGMP levels results in the activation of K^+^ channels. A link between the activation of the cGMP/G kinase pathway and the opening of BK_Ca_ channels has been well characterized [14], [15], and there is also evidence, albeit less well documented, that this second messenger system can activate K_V_ [16], [17], K_IR_, [17] and K_ATP_ [17], [18], [19] channels in vascular smooth muscle.

Glabridin might increase [cGMP] either by promoting its synthesis or by inhibiting its breakdown, e.g. by acting as a PDE inhibitor. In the former case, it would be predicted that inhibition of PDE would not prevent, or would enhance, glabridin's actions, whereas in the latter one might expect that glabridin would have no effect. We observed that following treatment of mesenteric arteries with 200 μM IBMX, glabridin was no longer able to cause relaxation. Moreover, the combination of glabridin and IBMX caused an increase in tissue [cGMP] which was similar to that induced by IBMX alone. This concentration of IBMX would be expected to fully block PDE5 [20], the main PDE involved in metabolizing cGMP in the vasculature [21]. These data therefore are consistent with the possibility that glabridin is acting as a PDE inhibitor.

A recent comprehensive review of the pharmacodynamics and pharmacokinetics of glabridin [2] has highlighted the fact that glabridin's antioxidant properties, which are thought to be responsible for many of its other biological effects, have been observed in studies employing micromolar concentrations, whereas its plasma concentrations are likely to be closer to 10 nM. On the other hand, concentrations of glabridin in the nanomolar range stimulated estrogen receptors [3], leading Simmler et al. to suggest that, of all the documented effects of glabridin, its estrogenic properties were most worthy of further study [2]. As stimulation of estrogen receptors has been shown to cause acute vascular relaxation via a mechanism which is dependent on cyclic GMP and BK_Ca_ channels [22], [23], [24], [25] we examined whether the vasorelaxation to glabridin was blocked by the estrogen receptor antagonist ICI 182780, and found that it was not. However the lack of effect of ICI 182780 did not provide definitive evidence against a role for estrogen receptors, because although this drug was observed to block 17β-estradiol -induced vasorelaxation in some arteries [19], [26], it was reported to have no effect on the relaxation to either 17β-estradiol or the selective estrogen receptor modulator raloxifene in rat superior mesenteric arteries [25]. Chan and colleagues similarly showed that dilation by raloxifene of third-order rat mesenteric arteries was insensitive to ICI 182780, and that this effect of raloxifene was almost completely suppressed by L-NAME [27]. However, we saw no effect of L-NAME on the response to glabridin. More importantly, raloxifene was shown by Tsang et al. (2004) to relax depolarization-induced contractions in rat cerebral arteries [28], and whereas we similarly found that raloxifene markedly attenuated the contraction evoked by 80KPSS in rat mesenteric arteries, glabridin had no effect on the high K^+^ contraction. On balance, therefore, the results with ICI 182780 and raloxifene imply strongly that glabridin was not causing vasorelaxation by stimulating estrogen receptors.

Glabridin concentrations in the plasma following ingestion of licorice root-containing preparations are not known. Aoki et al., (2007) estimated steady state plasma glabridin concentrations were between 0.6 and 2.4 ng/ml (2–7 nM) in healthy human subjects who ingested 1200 mg of licorice flavonoid oil (LFO; contained ~ 12 mg of glabridin) daily for several weeks, a dose which was apparently tolerated with no ill effects [7]. Tominaga et al., 2009 used a similar dose (900 mg daily) to show in a randomized trial that LFO caused a significant reduction in BMI and visceral fat [4]. Although we did not attempt to examine the effect of glabridin applied on its own at concentrations below 0.1 μM, its application at concentrations of 10 and 30 nM caused further vasorelaxation when added in the presence of 100 nM SNP. It therefore seems that glabridin is able to exert measurable acute effects on vascular tone at plasma concentrations close to those which could occur in individuals ingesting glabridin-enriched foods or supplements.

In conclusion, our results indicate that glabridin induced a vasorelaxation of rat mesenteric arteries which was associated with the opening of K^+^ channels and a concomitant rise in tissue cGMP levels. Glabridin was not able to cause vasodilation, or a further rise in cGMP, in the presence of the non-selective PDE inhibitor IBMX, consistent with the possibility that it was acting as a PDE inhibitor. In the presence of SNP, a measurable vasorelaxing effect of glabridin was observed at nanomolar concentrations, implying that glabridin may be able to influence vascular tone at plasma concentrations which might be attained following its consumption, particularly in the presence of another drug which has raised cGMP levels. Verification of the relevance of these findings to the use of glabridin as a food supplement or an aid to weight loss [4] must, however, await future studies in humans.

## Conflict of interest

None.

## References

[1] Saitoh T., Kinoshita T., Shibata S. (1976). New isoflavan and flavanone from licorice root. Chem. Pharm. Bull..

[2] Simmler C., Pauli G.F., Chen S.N. (2013). Phytochemistry and biological properties of glabridin. Fitoterapia.

[3] Somjen D., Knoll E., Vaya J., Stern N., Tamir S. (2004). Estrogen-like activity of licorice root constituents: glabridin and glabrene, in vascular tissues in vitro and in vivo. J. Steroid Biochem. Mol. Biol..

[4] Tominaga Y., Nakagawa K., Mae T., Kitano M., Yokota S., Arai T., Ikematsu H., Inoue S. (2009). Licorice flavonoid oil reduces total body fat and visceral fat in overweight subjects: a randomized, double-blind, placebo-controlled study. Obes. Res. Clin. Pract..

[5] Gupta P.K., Subramani J., Singh T.U., Leo M.D., Sikarwar A.S., Prakash V.R., Mishra S.K. (2008). Role of protein kinase G in nitric oxide deficiency-induced supersensitivity to nitrovasodilator in rat pulmonary artery. J. Cardiovasc. Pharmacol..

[6] Aaronson P.I., Benham C.D., Evans J.M., Hamilton T.C., Longman S.D., Stemp G. (1996). Potassium channel electrophysiology in vascular smooth muscle cells and the site of action of potassium channel openers. Potassium Channels and Their Modulators.

[7] Aoki F., Nakagawa K., Kitano M., Ikematsu H., Nakamura K., Yokota S., Tominaga Y., Arai N., Mae T. (2007). Clinical safety of licorice flavonoid oil (LFO) and pharmacokinetics of glabridin in healthy humans. J. Am. Coll. Nutr..

[8] Fuhrman B., Buch S., Vaya J., Belinky P.A., Coleman R., Hayek T., Aviram M. (1997). Licorice extract and its major polyphenol glabridin protect low-density lipoprotein against lipid peroxidation: in vitro and ex vivo studies in humans and in atherosclerotic apolipoprotein E-deficient mice. Am. J. Clin. Nutr..

[9] Rosenblat M., Belinky P., Vaya J., Levy R., Hayek T., Coleman R., Merchav S., Aviram M. (1999). Macrophage enrichment with the isoflavan glabridin inhibits NADPH oxidase-induced cell-mediated oxidation of low density lipoprotein. A possible role for protein kinase C. J. Biol. Chem..

[10] Yehuda I., Madar Z., Szuchman-Sapir A., Tamir S. (2011). Glabridin, a phytoestrogen from licorice root, up-regulates manganese superoxide dismutase, catalase and paraoxonase 2 under glucose stress. Phytother. Res..

[11] Lee J.W., Choe S.S., Jang H., Kim J., Jeong H.W., Jo H., Jeong K.H., Tadi S., Park M.G., Kwak T.H., Man K.J., Hyun D.H., Kim J.B. (2012). AMPK activation with glabridin ameliorates adiposity and lipid dysregulation in obesity. J. Lipid Res..

[12] Bolton T.B., Lang R.J., Takewaki T. (1984). Mechanisms of action of noradrenaline and carbachol on smooth muscle of guinea-pig anterior mesenteric artery. J. Physiol..

[13] Knock G.A., Smirnov S.V., Aaronson P.I. (1999). Voltage-gated K^+^ currents in freshly isolated myocytes of the pregnant human myometrium. J. Physiol..

[14] Carrier G.O., Fuchs L.C., Winecoff A.P., Giulumian A.D., White R.E. (1997). Nitrovasodilators relax mesenteric microvessels by cGMP-induced stimulation of Ca-activated K channels. Am. J. Phys..

[15] Kyle B.D., Hurst S., Swayze R.D., Sheng J., Braun A.P. (2013). Specific phosphorylation sites underlie the stimulation of a large conductance, Ca(2 +)-activated K(+) channel by cGMP-dependent protein kinase. FASEB J..

[16] Tanaka Y., Tang G., Takizawa K., Otsuka K., Eghbali M., Song M., Nishimaru K., Shigenobu Koike K., Stefani E., Toro L. (2006). Kv channels contribute to nitric oxide- and atrial natriuretic peptide-induced relaxation of a rat conduit artery. J. Pharmacol. Exp. Ther..

[17] Dantas B.P., Ribeiro T.P., Assis V.L., Furtado F.F., Assis K.S., Alves J.S., Silva T.M., Camara C.A., Franca-Silva M.S., Veras R.C., Medeiros I.A., Alencae J.L., Braga V.A. (2014). Vasorelaxation induced by a new naphthoquinone-oxime is mediated by NO-sGC-cGMP pathway. Molecules.

[18] Kubo M., Nakaya Y., Matsuoka S., Saito K., Kuroda Y. (1994). Atrial natriuretic factor and isosorbide dinitrate modulate the gating of ATP-sensitive K^+^ channels in cultured vascular smooth muscle cells. Circ. Res..

[19] Hein T.W., Xu W., Kuo L. (2006). Dilation of retinal arterioles in response to lactate: role of nitric oxide, guanylyl cyclase, and ATP-sensitive potassium channels. Invest. Ophthalmol. Vis. Sci..

[20] Wang P., Wu P., Myers J.G., Stamford A., Egan R.W., Billah M.M. (2001). Characterization of human, dog and rabbit corpus cavernosum type 5 phosphodiesterases. Life Sci..

[21] Rybalkin S.D., Yan C., Bornfeldt K.E., Beavo J.A. (2003). Cyclic GMP phosphodiesterases and regulation of smooth muscle function. Circ. Res..

[22] Mugge A., Riedel M., Barton M., Kuhn M., Lichtlen P.R. (1993). Endothelium independent relaxation of human coronary arteries by 17 beta-oestradiol in vitro. Cardiovasc. Res..

[23] White R.E., Darkow D.J., Lang J.L. (1995). Estrogen relaxes coronary arteries by opening BK_Ca_ channels through a cGMP-dependent mechanism. Circ. Res..

[24] Alda J.O., Valero M.S., Pereboom D., Gros P., Garay R.P. (2009). Endothelium-independent vasorelaxation by the selective alpha estrogen receptor agonist propyl pyrazole triol in rat aortic smooth muscle. J. Pharm. Pharmacol..

[25] Keung W., Chan M.L., Ho E.Y., Vanhoutte P.M., Man R.Y. (2011). Non-genomic activation of adenylyl cyclase and protein kinase G by 17beta-estradiol in vascular smooth muscle of the rat superior mesenteric artery. Pharmacol. Res..

[26] Bucci M., Roviezzo F., Cicala C., Pinto A., Cirino G. (2002). 17-beta-oestradiol-induced vasorelaxation in vitro is mediated by eNOS through hsp90 and akt/pkb dependent mechanism. Br. J. Pharmacol..

[27] Chan Y.C., Leung F.P., Wong W.T., Tian X.Y., Yung L.M., Tsang S.Y., Yao X., Chen Z.Y., Huang Y. (2010). Therapeutically relevant concentrations of raloxifene dilate pressurized rat resistance arteries via calcium-dependent endothelial nitric oxide synthase activation. Arterioscler. Thromb. Vasc. Biol..

[28] Tsang S.Y., Yao X., Essin K., Wong C.M., Chan F.L., Gollasch M., Huang Y. (2004). Raloxifene relaxes rat cerebral arteries in vitro and inhibits L-type voltage-sensitive Ca^2 +^ channels. Stroke.

